# Bifurcations and the Emergence of L2 Syntactic Structures in a Complex Dynamic System

**DOI:** 10.3389/fpsyg.2020.574603

**Published:** 2020-10-29

**Authors:** D. Reid Evans, Diane Larsen-Freeman

**Affiliations:** ^1^Office of Graduate Medical Education, University of Massachusetts Medical School, Worcester, MA, United States; ^2^Department of Linguistics, School of Education, University of Michigan, Ann Arbor, MI, United States

**Keywords:** complex dynamic systems theory, bifurcations, fractals, L2 development, accuracy, fluency, non-linearity

## Abstract

We report on a complex dynamic systems study of an untutored adult French learner’s development of English syntax, specifically two non-finite adverbial constructions. The study was conducted over one academic year of 30 weeks. From an analysis of L2 speech samples collected weekly, certain patterns in the flux emerged. The learner’s ensuing second language development is characterized by a series of bifurcations, stemming from forms competing for the same functional terrain. Each bifurcation is accompanied by turbulence as the system moves from one attractor state to another. The transition is characterized by loss of stability, an increase in variability, and a period of dysfluency. It is in the dynamic relationship of accuracy and fluency that novel syntactic forms emerge, both convergent with and divergent from dominant contextual patterns, with dominance established by consulting a well-known corpus of contemporary English. Non-linear development occurs with continuous and iterative exposure to and interaction in English—from relexification to adaptation and synchronization, animated by the learner’s perception and memory of regular sequential associations, to pruning of divergent forms. What results over time is a branching hierarchy, connecting online processing with over time development. Multiple competing forms continue to co-exist in the learner’s repertoire, which is likely more typical of adult L2 development than of L1 acquisition.

## Introduction

Emergentism has been a powerful conceptual framework adopted in many scholarly arenas, although it has been interpreted somewhat differently among these. For the purposes of this issue of *Frontiers in Psychology*, we consider emergence to mean the arising of linguistic structures from patterns of usage over time (MacWhinney, 2015). In this article, which deals with L2 or second language learning, we call upon one approach to investigating emergentism, namely complex dynamic systems theory (CDST). CDST considers the complexity of the language system to be derived from the dynamic interaction of its many interdependent subcomponents, and they with the context in which language is used. CDST also characterizes language learning as a multidimensional process—involving embodied cognitive, affective, social, and neurological factors, all operating within a given context (Larsen-Freeman and Cameron, 2008).

Through a careful analysis of data collected during a 30-week longitudinal study of an untutored adult male French learner of English (Evans, 2019), we are able to identify “patterns in the flux” (Larsen-Freeman, 2017)—patterns that emerge *while* the learner’s system^1^ transitions to new levels of grammatical complexity. Given CDST’s processual orientation, we give special attention to a series of bifurcations that characterize the learner’s development of non-finite adverbial constructions. The learner’s developmental trajectory starts off with a relexification^2^, an obvious transfer from the learner’s L1 French. With continued exposure to English, and because the learner is motivated to participate in the dominant social group, he notices a discrepancy between what he is producing and what he perceives through experience, in so doing making an inference which triggers the first bifurcation in his L2 development. However, the fact that L2 learners exhibit reduced sensitivity to competing alternatives, possibly due to limited attentional resources, means that the more contextually dominant^3^ form is not always immediately selected (Tachihara and Goldberg, 2020).

The bifurcations emerge as a result of the competition between forms for the same functional terrain. Each bifurcation is accompanied by turbulence as the system moves from one attractor state to another. The transition is characterized by the loss of stability, an increase in variability, and a period of disfluency. Novel syntactic forms emerge, both convergent with and divergent from dominant contextual patterns, the dominance attested to by corpus data. Through adaptation and social synchrony with English speakers (Larsen-Freeman, 2020) and iterative exposure to and use of the target language by the learner, novel L2 forms multiply, animated by the learner’s perception and memory of regular sequential associations. Notably, the bifurcations are not one-off phenomena; instead, they occur in an iterative, cladistic series. Following the example of synaptic pruning in neuronal systems (Webb et al., 2001), we propose the mechanism of pruning to explain how linguistic representations slowly “prune” from multiple representations in the L2 learner’s repertoire. We also look to the system’s hysteresis, e.g., the entrenchment of the L1 representation, the stochastic environment, and the heterogeneity of linguistic competence to explain why competitors continue to coexist at the neuronal level.

In fact, certainly in adult L2 development, it is not that the less common form disappears forever. Thus, even though the contextually dominant form may win out over the others by becoming the more prevalent form in the learner’s repertoire, and thus restoring stability to the learner’s system, it is not that the system ever completely settles down. CDST places great stock in the influence of the context. The interaction of the system under construction/use and context is invoked to explain the reappearance of less favored options under certain contextual conditions/constraints. In other words, there is no end state to language learning (Larsen-Freeman, 2006a).

### Complex Dynamic Systems Theory

Since its introduction to the field of applied linguistics (Larsen-Freeman, 1997), CDST has gained increasing favor among those whose interests lie in understanding second language use and development as an emergent, non-linear process. Indeed, language development viewed from the perspective of CDST accords well with dynamic systems emergentist approaches (van Geert and Verspoor, 2015) and provides a useful lens through which to view emergent linguistic phenomena. Such value derives, in part, from CDST’s processual approach to the study of language development (Lowie and Verspoor, 2015), placing greater emphasis on the *process* by which language emerges and not on the endpoint of acquisition (de Bot, 2015).

Notably, adopting the process-oriented approach championed by, although not exclusive to, CDST has allowed researchers to capture the dynamism of language development as it unfolds over time. As MacWhinney (2006) rightfully cautions, “emergentist explanations must explain where a linguistic behavior comes from. It is not enough to point to the complexity of some linguistic behavior and to declare that it must be emergent” (p. 732). The robust theoretical (Larsen-Freeman and Cameron, 2008, Larsen-Freeman, 2017) and methodological (Verspoor et al., 2011; Hiver and Al-Hoorie, 2020) treatments of CDST have offered complexity researchers the tools to meet this challenge head on. With its emphasis on tracing the emergence of language longitudinally across dense, closely-spaced measurements, researchers are able to view development retrodictively, that is by tracing change backward through time (Dörnyei, 2014). In doing so, not only do complexity-informed studies seek the antecedents of emergent linguistic behavior as MacWhinney (2006) suggests, but, taken a step further, they uncover the unique ways in which the interdependent constructs interact to promote the emergence of increasingly complex linguistic behavior.

From a CDST orientation, it is precisely this approach to the study of emergence that paints a more complete picture of development. In many traditional, product-oriented studies, developmental outcomes were limited to one or few independent variables that were most frequently measured independently at fixed moments in time. This approach proves problematic when working with human subjects as controlling for linguistic and psychological factors one at a time is difficult, often unacceptable to the learner, and leads to spurious interpretations. As a relational theory, alternatively, CDST places heightened emphasis on a more holistic view as “one cannot fully understand one part of a complex system if one does not look at its relationship with another” (Larsen-Freeman, 2020, p. 190). Thus, interdependence within the developing system takes precedence. For this reason, emergentist accounts of second language development warrant greater attention to the dynamic interaction of multiple constructs. Such dynamic interaction analysis has surfaced in a handful of complexity-informed studies (e.g., Hepford, 2017; Evans, 2019; Yu and Lowie, 2019) and has offered insight into the ways in which linguistic constructs come together to both support or constrain development (van Geert, 1994).

### CDST and Patterns in the Flux

Germane to the behavior of complex systems is the tendency to exhibit emergent underlying patterns—i.e., patterns in the flux—as the system self-organizes toward growing complexity. If emergence in language development is taken as the arising of linguistic structures from patterns of usage over time (MacWhinney, 2015), evidence of spontaneous pattern formation (van Geert, 2008) within the linguistic system may provide valuable insight into the process of emergence and the complexity that ensues. Indeed, at critical moments in time, complex systems experience abrupt, qualitative shifts from one discernable pattern of behavior to another (Kelso, 2009). It is at these precise moments of phase transition, or “points of instability and turbulence where old patterns break down and new ones appear” (Lewis, 2000, p. 39), that increasingly disordered, entropic behavior makes way for new attractor states, or “pockets of stability” (Hiver, 2015, p. 21) to emerge. Thus, seeking to understand how the interconnected components of the complex linguistic system converge to give rise to new patterns of behavior has become the crux of the CDST agenda (Larsen-Freeman and Cameron, 2008).

In sum, human language, in both its development and use, is now widely accepted as a complex adaptive system (Ellis and Larsen-Freeman, 2009). With this appellation, undoubtedly, come new challenges and new approaches to its study. One such challenge, of course, is to move beyond descriptions of the static phases of development, instead focusing on the transition between such phases (de Bot et al., 2013) and the ways in which patterned language behavior emerges in context. As complex systems are known to behave in distinct ways, language researchers committed to a CDST view must foreground the unique behavior of complex systems focusing on non-linearity and the patterns in the flux that characterize language development. In what follows, we highlight one particular pattern in the flux—the bifurcation—while paying special attention to the interaction of fluency and accuracy at these unique points of transition. In doing so, we gain insight not only into the emergent patterns of development, but equally into the ways in which competition between syntactic constructions motivates such transitions.

## Materials and Methods

Commensurate with a CDST theoretical orientation, in this study we adopt a longitudinal design which allows a particular unit of analysis to be followed over close, densely-spaced intervals for a given period of time (Hilpert and Marchand, 2018). Once a dataset is complete, data analysis is said to progress retrodictively, that is, by a method in which principal findings are identified and then traced backward through the dataset to identify the factors or patterns which have given rise to the changes within the system (Dörnyei, 2014). The complexity approach to study design and data analysis is fruitful for emergentist accounts of language development as consecutive measurement of specific constructs permits researchers to capture the unfolding of emergent linguistic phenomena over time.

### Participant

The participant in this study, Alceste, was a 27-year-old untutored learner of English as a foreign language. From the Francophone region of Switzerland, Alceste had come to the United States via an exchange program with an assignment to teach university-level French for 1 year at a large public university in the Northeast. Upon arrival, initial approximation of Alceste’s English proficiency based on conversational and narrative data placed him at the intermediate low level (American Council on the Teaching of Foreign Languages [ACTFL], 2012). His linguistic production at this time was characterized by frequent false starts, repetitions, and abandoned utterances. During initial data collection, Alceste frequently asserted his concern that his strong accent limited his comprehensibility with native speakers and suggested that improving his accent was a strong goal while in the United States. Although Alceste did not enroll in any formal instruction in English as a second language during his sojourn, his eagerness to learn English motivated him to seek out opportunities to interact with English speakers in addition to his daily interactions with students and colleagues.

### Data

Data for the present study came from two distinct tasks designed to collect oral production data on a weekly basis for one academic year. Performance undoubtedly differs across oral tasks given, among other things, the disparate nature of dialogue vs. monologue (Michel et al., 2007). As such, Alceste was asked to complete both a monologic narrative and a dialogic conversation task each week to capture a more comprehensive range of his oral proficiency. The narrative task consisted of recounting a movie or television show that he had seen or a book that he had read that particular week. Beyond these minimal specifications, the choice of prompt was not controlled in any way, given that prompt choice has been reported to have little effect on measures of grammatical complexity and accuracy (De Jong and Vercellotti, 2016). Task duration for the monologic narrative was approximately five minutes each week. Similarly, weekly conversations between Alceste and the researcher were recorded and, although not scripted in any way, recurrent topics were common such as his position as an instructor of French, his interest in French literature, cultural differences between the United States and Switzerland, and his life in the Northeast. Weekly conversations lasted for a minimum of 20 minutes each week, though frequently Alceste’s desire for prolonged interaction allowed for lengthier interactions.

Choices as to the duration and density of data collection were given the following consideration. As the participant remained in the United States for just one academic year, data collection began and ended with his arrival and departure, respectively. Though density of data varies greatly in CDST studies of L2 development, a weekly timescale was established to provide a fine-grained account of development without the imposition of daily or even bi-weekly data collection. To be sure, had data collection begun or ended at alternate moments in time, or had it progressed at more random or lengthy intervals, the emergence of the two syntactic structures detailed in this article may have been obscured. Similar, yet less frequently used, syntactic constructions (e.g., *instead of* + ing), were evident throughout the dataset, though the density and duration of data collection did not allow for bifurcations in their trajectories to be captured.

### Data Analysis

Transcribed oral production data from both tasks were first segmented into analysis of speech units (AS-units), a widely used measure in L2 oral text analysis. Minimally defined as any “independent clause, or sub-clausal unit, together with any subordinate clause(s) associated with either” (Foster et al., 2000, p. 365), the AS-unit allows for focused attention on hypotaxis as subordinate structures are emphasized in this analysis. In our analysis we drew on the constructs of accuracy and fluency to provide an understanding of the development of one particular syntactic structure—the non-finite adverbial clause, *before*-headed and *without*-headed adverbial clauses, in particular. The measures adopted for this analysis are discussed below.

#### Accuracy

Distinctions between global and local measures of accuracy are common in studies applying this construct as each may capture development in different ways (Foster and Wigglesworth, 2016). Broadly speaking, global measures of accuracy count all erroneous forms within a dataset and are displayed as ratios or proportions of errors per a given linguistic unit (e.g., errors per 100 words; errors per AS-unit). Local measures, on the other hand, are more selective and focus on specific constructions most often related to syntax. As this study focused specifically on the development of non-finite adverbial clauses, the measure of accuracy was local in nature.

Indeed, the construct of second language accuracy has been questioned from a CDST perspective, with proponents calling for a more situated understanding of what constitutes “accurate” production (Larsen-Freeman and Evans, 2019). This idea, paired with the emergent synchrony that characterizes language use in social contexts (Larsen-Freeman, 2020), motivated us to consider accuracy in more ecological terms. Thus, we sought, instead, to establish the language user’s convergence and/or divergence from L2 usage patterns, making use of a widely cited linguistic corpus. To determine contextually convergent vs. divergent forms, word sequences were evaluated using the COCA corpus (Davies, 2008). Following Larsen-Freeman (2015), phrases appearing in the dataset were cross-referenced with the corpus and part-of-speech tags were used to allow for broad lexical variation within phrases. A threshold type frequency of two tokens was selected as minimal evidence that a phrase was contextually convergent. Those phrases returning fewer than two tokens were considered non-dominant, that is, that their form did not converge with the typical patterns of production in the language use ecology. A low frequency threshold of two tokens gives the benefit of the doubt to the speaker further mitigating researcher subjectivity (see Table 1).

**TABLE 1:** COCA search parameters and token frequencies.

**Phrase in dataset**	**Partial search**	**Token frequency**	**Convergent**
*Before to come*	Before_i TO VB0	0	NO
*Before starting*…	Before_i VVG	56,567	YES
*Without want*…	Without_i VB0	0	NO
*Without explaining*	Without_i VVG	59,014	YES

**_*i, preposition; TO, infinitive marker; VB0, base form; VVG, -*ing* participle.*

#### Fluency

Fluency in oral production is defined as “the speed and efficiency with which [learners] can access and implement relevant L2 information to communicate meanings in real time” (Housen et al., 2012, p. 6). In our data, we included several measures of fluency that were associated with the production of non-finite adverbial clauses. These included measures of breakdown fluency, namely silent pauses and filled gaps, and repair fluency at those moments in which Alceste engaged in self-repair. Transcription conventions are displayed in Table 2 below.

**TABLE 2:** Transcription conventions.

**Symbol or format**	**Description**
Upright slash (|)	AS-unit boundary
Double colon (::)	Clausal boundary
Brackets {}	Self-repair
(.)	Unfilled pauses
(&)	Filled pauses

#### Bifurcation Analysis

**FIGURE 1:**
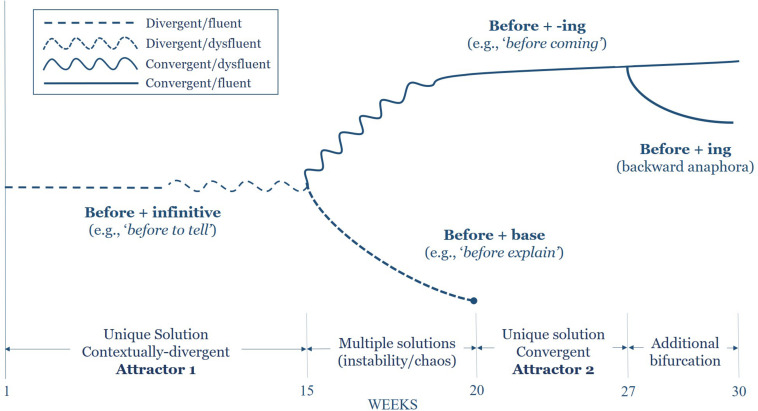
Bifurcation region of before-headed non-finite adverbial clauses.

**FIGURE 2:**
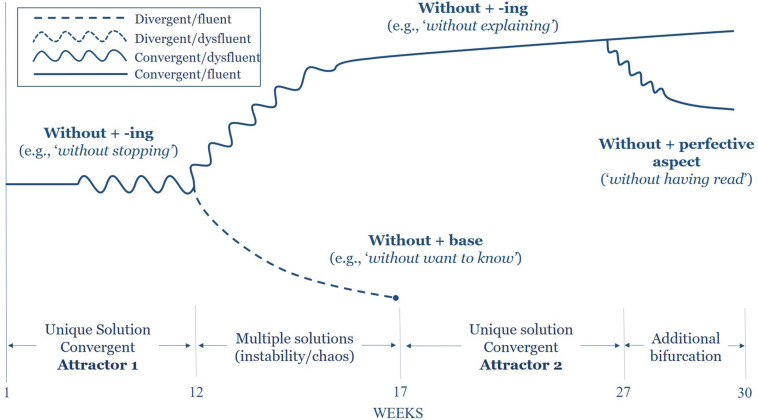
Bifurcation region of without-headed non-finite adverbial clauses.

Data analysis leading to the bifurcation diagrams shown in the section “Findings” below proceeded retrodictively. Once data collection was complete and clear developmental changes were identified in both *before*- and *without*-headed adverbial constructions, all occurrences of these forms were extracted from the dataset along with the concomitant accuracy and fluency of production. Next, the development of these forms was traced backward through the dataset by plotting each adverbial construction in its temporal order of appearance. This process illustrated the bifurcated trajectories of development as novel forms appeared, co-existed, and either remained or were pruned from the dataset. When plotted visually to include the associated accuracy and fluency of production, these trajectories clearly depict the bifurcations visible in Figures 1, 2 below.

### Findings

The progressive development of non-finite adverbial clauses, when viewed in conjunction with measures of accuracy and fluency, sheds light on the critical relationship among these constructs in the developing L2 linguistic system. While non-finite adverbial clauses may take many forms, from the beginning, those clauses with -*ing* verb forms proved challenging for Alceste. In particular, many non-finite adverbial clauses headed by prepositions (e.g., *before, without*, and *about*) were produced erroneously, yet appeared fluent as no dysfluency features were present in their production. This was overtly apparent in the *before* + infinitive constructions produced consistently throughout the beginning weeks of data collection and used to express an action prior to that of the matrix clause. These are evidenced in (1a) and (1b) below:

(1a) Alceste: | I really want :: to {lost} lose my accent at least a little bit | and (&) to don’t have :: to think :: *before to talk*|(Conversation – Week 1)
  (1b) Alceste: | I want :: to be sure :: *before to tell her*| (Conversation – Week 5)

Here, the *before*-headed structures in the English examples above are analogous to those found in Alceste’s native French as French relies on infinitive forms in constructions conveying similar semantic information. *Before talking*, for example, is expressed by *avant de* followed by the infinitive *parler*. Formulated in this way, Alceste’s first attempts with this construction appear to be a relexification, the influence of his native French, as he produced these clauses from the outset in the manner typical of his L1.

Yet limiting analysis strictly to putative relexification paints only a partial picture. Importantly, the first several occurrences of the sentence final *before* + infinitive constructions were uttered confidently and fluently as any dysfluency features relating to the articulation of these clauses were notably absent from Alceste’s speech. As complex systems frequently find themselves in attractor states, or any discernable pattern representing a “pocket of stability” (Hiver, 2015, p. 21), it seems as though the fluency with which these forms were produced at the outset of data collection may point to the initial attractor state of the system, i.e., one presumably shaped by the L1 pattern.

It was not until week nine, however, that the initial attractor constraining the system began to destabilize, evidenced by the growing dysfluency in production. During one conversation, the topic of discussion turned to the laws regarding alcohol use in both Switzerland and the United States. It was at this point that he suggested the following:

(2) Alceste: | Yeah so you can drink a beer :: *before to drive* (.) *{could drive}*| (Conversation – Week 9)

In this example, the independent clause *you can drink a beer* is followed first in the manner characteristic of production until this point, yet after a brief hesitation (.), Alceste attempts to self-correct with the phrase *could drive*. As the first instantiation of dysfluency related to *before-*headed clauses, the appearance of the dysfluency features noted above is telling. Taken together, the presence of both breakdown and repair fluency at the time of articulation suggests that, at least to some extent, Alceste may have been aware that the form of this construction did not align with the language usage patterns in the environment. Looking forward, the attractor state governing the production of these structures was nearing a moment of criticality as these adverbial constructions would soon undergo a qualitative change. Of course, as complex dynamic systems are feedback sensitive, the ecological pressure from the English-speaking context may have engendered the ensuing development toward more contextually dominant forms of expression.

Finally, at week 16, the first occurrence of a contextually dominant *before*-headed clause was evidenced in Alceste’s speech. While discussing his frequent early arrival to campus, he stated the following:

(3) Alceste: | I can just read a little bit :: (.) *before starting the class*| (Conversation – Week 16)

Prior to this utterance, Alceste had not produced a target-like *before* clause in a manner consistent with the L2 ecology. Once again, fluency of production plays an important role in this example as the silent pause (.) indicates that Alceste may have used this brief instant as a moment of online planning to retrieve the contextually dominant form for the first time that the data captured it. Perhaps his role as an instructor, with heightened attention given to the idea of “starting class,” motivated the contextually dominant form to outcompete those forms which had previously dominated production.

Curiously, as many theories of language acquisition attest, the emergence of contextually dominant *before* + *-ing* clauses did not follow a fluid, linear progression. In fact, after transitioning to the target-like *before* + *-ing* construction for the first time at week 16, an alternative form emerged in the dataset and, for approximately 4 weeks, existed in direct competition with the target-like construction. An example is provided in (4) below:

(4) Alceste: | he didn’t experienced everything :: *before explain {them} them (.) through the literature* | (Conversation – Week 20)

In this instance, Alceste makes use of a competing, contextually non-dominant *before* + *base form* construction that had not materialized previously in the data. Notably, we see that he has learned to drop the infinitival marker “to,” though control of the –ing form still seems to be out of grasp. Indeed, ephemeral language forms are typical in L2 learner data (Larsen-Freeman, 2006b), yet as both convergent and divergent forms were consistently present between weeks 16 and 20, the bimodality seen during this timeframe points to competition between major form alternatives (MacWhinney, 2001). From a CDST viewpoint, such bimodality, understood as two potential states or equilibria within a behavior (Ruhland and van Geert, 1998), is characteristic of a transition from one state to another. This complete emergence of *before*-headed clauses is illustrated in Figure 1 above.

Figure 1 demonstrates the emergence of contextually dominant *before*-headed non-finite adverbial clauses in Alceste’s speech production over one academic year. Importantly, several key features of this model must be clarified to allow for the appropriate interpretation of this figure. To begin, the dashed lines visible toward the beginning of the trajectory mark the contextually divergent nature of the forms produced during these periods, whereas the solid lines represent convergent forms that emerged as data collection progressed. Furthermore, the oscillating lines visible between weeks 9 and 22 are indicative of the dysfluency features that were present in the production of these structures (viz., silent pauses, filled gaps, and self-repair). Oscillating dashed lines represent dysfluent, contextually divergent forms; oscillating solid lines represent dysfluent contextually convergent forms. Visual interpretation of the phenomenon in this way readily evinces the bifurcation that occurred in the emergence of these forms as well as the concomitant dysfluency that accompanied this marked divergence.

From a complexity perspective, this model allows us to identify the apparent attractor states that governed the production of these grammatical forms and, most significantly, to illustrate the particular ways in which accuracy and fluency converged during this transition. Although initially fluent in their production, the contextually non-dominant *before* + infinitive (e.g., *before to come*) constructions quickly entered a period of instability as the first attractor state moved away from equilibrium. Typical of the behavior of complex systems, the ensuing destabilization was marked with increasing variability in fluency. In what followed, this instability increased to a point at which a significant bifurcation occurred at week 16 and, for a period of roughly 4 weeks, resulted in the competition of major form/meaning alternatives. Referring once again to Figure 1 above, we note the oscillation in the line representing the first 4 weeks of target-like *before* + *-ing* constructions. The importance of this period cannot be understated. As both multiple competing forms existed during these 4 weeks with varying degrees of fluency, this transition period is marked by the inherent instability within the incipient linguistic system. From this chaotic period, however, through the language usage patterns to which Alceste was exposed, new order emerged in the form of a contextually dominant syntactic construction.

Highlighted in Figure 1 above, the progressively increasing stability of the second attractor state engendered a further bifurcation at week 27, resulting in a branching hierarchy, much as in a cladistic taxonomy. At this moment, Alceste produced the expression *before even reading it* in the sentence-initial position with no associated disfluency features. As a milestone of linguistic development, the instantiation of this combinatorial structure is significant in that the *before* clause introduces a more complex fronted, referentially dependent null element that appears before the subject NP—a phenomenon known as backward anaphora. This is expressed in (5) below (the dependency is denoted with *_*i*_*).

(5) Alceste: | before even *_*i*_* reading it :: when I *_*i*_* hear that… I’m *_*i*_* like wow | (Conversation – Week 27)

Highlighting the interdependence of the complex linguistic system, the bifurcation in the emergence of *before*-headed adverbial clauses illustrates the role that accuracy and fluency play in the transition between the attractor states governing syntactic forms. In this way, the self-organization of complex syntax, motivated by the ecological pressure of the L2 environment, is marked by destabilization in the fluency of production along with the emergence of both contextually convergent and divergent forms. Ultimately, as the underlying grammatical structures self-organize to align with the L2 ecology, the patterns available to the learner serves to reinforce contextual convergence.

### *Without*-Headed Non-finite Adverbials

Similar to the *before*-headed constructions outlined above, those non-finite adverbials introduced by the preposition *without*, meaning the absence or lack of something, present an equally unique developmental trajectory in Alceste’s emerging L2 (see Figure 2). Curiously, both non-finite *before* and *without* clauses are morphosyntactically isomorphic in that these prepositions combine with –*ing* verb forms, yet their development proved to be somewhat distinct. Although the initial occurrence of this form was indeed convergent at week 3 [see (6) below], the ensuing dysfluent and/or divergent forms produced in the coming weeks were indicative of the inherent instability within Alceste’s linguistic system.

(6) Alceste: | he was writing :: *without stopping too* |(Conversation – Week 3)

In contrast to the *before*-headed adverbials discussed above, production of *without-*headed clauses at the beginning of data collection was convergent, yet these utterances quickly destabilized and wavered between convergent and divergent forms with increasing dysfluency as the weeks progressed. At week 8, Alceste produced a target-like, yet dysfluent *without* clause demonstrating heightened breakdown fluency as two syntactic forms competed for functional terrain.

(7) Alceste: | I like the fact :: that you can speak with somebody in Spanish :: *without (.) {to} being in a class* | (Conversation – Week 8)

As the conversation turned to the weekly Spanish roundtable held within the university’s Romance language department, Alceste included the sentence-final adverbial clause to emphasize the non-credit-bearing nature of these dialogues. As seen in (7) above, this clause contains the co-occurrence of both breakdown and repair fluency. In a sense monitoring his production, it appears as though after first initiating the clause, Alceste hesitated for a moment, caught himself as he produced the erroneous infinitival marker *to*, then abandoned this construction in favor of the target-like *being in a class*. Although the clause is ultimately produced accurately, further examination of this utterance points to a moment in which conflicting (bimodal) syntactic knowledge leads to a breakdown in fluency.

Subsequent to the utterance in week 8, Alceste continues to vacillate between both convergent and divergent *without* clauses. Most striking, however, is the change that is noted in his speech at week 12 and that remains present until week 17 as Alceste begins to directly mirror the bifurcation apparent in his *before* clauses by producing target-deviant *without* + base form constructions. These are demonstrated in (8a), (8b), and (8c) below.

(8a) Alceste: | you have :: to make sense :: *without even (.) read the book* | (Conversation – Week 12)
(8b) Alceste: | he decides :: to just (.) {run} go running for three years :: (&) *without stop* | (Narrative – Week 13)
(8c) Alceste: | yeah understand it :: *without (.) explain the language* | (Conversation – Week 17)

The three examples provided here, all of which were articulated with some degree of dysfluency, mirror the form produced in the *before*-clause bifurcation noted in the previous section. It seems that, at first, the production of *before-* and *without-*headed clauses was governed by an item-based analysis; yet, through continued exposure, as the two structures converge, Alceste is able to extract higher-level patterns. Furthermore, this process equally highlights the interdependence of the internal forms as the *before* clauses ostensibly occasion a regressive effect on the *without* clauses.

Once again, much in the same way as the trajectory of *before*-headed clauses discussed above, an additional bifurcation was noted in the production of *without*-headed clauses, though with distinct grammatical structure. At week 27, nearing the end of his sojourn in the United States, Alceste adds to his repertoire *without* adverbial clauses including perfect participle predicates, thus expanding the meaning-making potential of his system. Though still non-finite in nature, the perfect participle is constructed with two distinct non-finite verbs as demonstrated in (9). Perhaps the increasing stability of the contextually dominant structure allowed Alceste to extend his proficiency with these forms to include aspectual information which is not expressed in the *without* + *-ing* form alone.

(9) Alceste: | you cannot have a PhD in French literature :: *without (.) having read at least one of his novels* | (Conversation – Week 27)

By this point, the contextually divergent preposition + base form constructions present in both *before-* and *without-*headed clauses had precipitated out of Alceste’s language production. The new-found stability of the second attractor state had thus produced new levels of equilibria within the system to an extent that both fluent and accurate forms were ubiquitous within the data. Most notably, the move at week 27 toward higher levels of complexity via *without* + *perfect participle* clauses co-occurred with the novel flexibility of *before* adverbial clauses to appear in sentence initial position, and once again, the branching pattern is noted with the onset of the second bifurcation.

## Discussion

In this article, we have illustrated the patterns of emergence of two distinct, yet related non-finite adverbial constructions as competition for semantic space spawned bifurcations in their development. In doing so, the process-oriented nature of CDST research (Lowie, 2017), with its emphasis on the relationship of accuracy and fluency in the developing linguistic system (Larsen-Freeman, 2020), has allowed us to identify the patterns in the flux and how these contribute to the self-organization and emergence of complex syntactic forms.

Such a dynamic process, as argued from the outset of this article, is amenable to the complexity-informed perspective adopted here in that we easily note the non-linear nature of language development rife with increasing instability and points of divergence, ultimately pushing the boundaries between stability and variability. Recognizing the significance of these moments of bifurcation as integral to the process of L2 development is not new (Plaza-Pust, 2008). However, a focus on heightened variability in accuracy and fluency as indicators of potential bifurcations certainly allows for a more fruitful analysis in the interpretation of dense longitudinal data.

In all, the data presented herein serve to accentuate the non-linear nature of L2 development as learning does not exist on a simple continuum of right and wrong, fluent and dysfluent, simple and complex. As has been noted in L2 research, outward developmental “regressions” may in fact be the essential elements from which true linguistic development can occur. In this way, these bifurcations operate similar to U-shaped patterns, where the increased variability in production eventually subsides and accuracy is restored. In the case of the bifurcations, however, we see that the picture is more complex. For one thing, the L2 competitors are not all internal to the system as is the case in the oft-cited U-shaped pattern found in the L1 and L2 learning of the regular and irregular past tense in English. Secondly, the pattern does not simply reflect a tension between accurate and inaccurate forms. Thirdly, bifurcations illustrate that even though novel forms appear in the learner’s repertoire often replacing or adding to previous forms, the competition between these forms does not simply vanish. The clear bimodality of production visible within a bifurcation diagram makes clear that the competition between forms is persistent and, in the case of L2 learners, such competition may produce regressions long after a contextually convergent form is learned. In sum, the transition from contextually divergent to contextually convergent is non-linear and cannot be conceived as a fluid transition between forms. The bifurcations illustrate the role that accuracy and fluency may play in pushing the development of syntactic forms from one attractor state to the next.

The analysis of adverbial constructions in Alceste’s oral production—specifically *before*- and *without*-headed clauses—illustrates the patterns of local interaction that emerge as the incipient linguistic system moves from one stable attractor state to another through apparent bifurcations in phase space (Prigogine and Stengers, 1984). For *before-* clauses specifically, this transition ensued according to the following sequence: (a) a stable, contextually divergent yet fluent form was consistently produced for several weeks; (b) the divergent form destabilized for a brief period indicated by the increasing attenuation of fluency; (c) at a critical point, a bifurcation occurred during which both a contextually convergent as well as a novel divergent form were produced; (d) finally, a new attractor state arose characterized by the accurate and fluent production of the syntactic structure, leading to (e) a second bifurcation in which a more complex syntactic structure emerged.

The bifurcation in non-finite adverbial constructions described above, in which the emergence of complex syntactic structures is understood in conjunction with accuracy and fluency, allows us to approach an imperfectly understood area of L2 development. Larsen-Freeman (2006b), in her discussion of the longitudinal trajectories of complexity, accuracy, and fluency in five Chinese learners of English, stresses that:

What one would like to know as an applied linguist is if any of [the variation presented in her data] is indicative of the bifurcations that signal the *instability* alluded to earlier, the instability that precedes a phase shift in the system (p. 611, emphasis added).

It seems as though the bifurcations in Alceste’s development of complex syntax would answer this question. Not only does the self-organization of underlying grammatical constructions result in a phase shift between attractor states, but equally we see that the periods of instability characterized by heightened dysfluency and bimodality of production are indelibly linked to this process. Seen in this way, language researchers interested in further pursuing investigation into bifurcations may benefit from greater attunement to the periods of (potentially anomalous) instability characteristic of stochastic systems.

Viewing language development as a series of bifurcations, however, leaves us with an equally important question. In their discussion of bifurcation phenomena, Prigogine and Stengers (1984) address the characteristic split in trajectories in which multiple solutions or states are available to a complex dynamic system. At these critical moments, the choice between following either of the two possible trajectories results from the competition of forces both internal and external to the system, and one trajectory frequently wins out over the other. Hence, as language researchers, our interest lies in understanding the competitive pressures which motivate the choice between trajectories when moments of bifurcation are reached. In the case presented above, Alceste’s developing linguistic system moved away from contextually non-dominant adverbial constructions toward the fluent production of forms aligned with the usage patterns in the L2 ecology. This move seems to be indicative of the influence of the properties of the external environment on the incipient language faculty (Larsen-Freeman, 1997; de Bot, 2015) as self-organization is often motivated by learners’ adaptation to the linguistic environments that surround them (Larsen-Freeman, 2006b) and to the behavior of social synchrony between interlocutors (Larsen-Freeman, 2020). Adaptation and social synchrony are presumably made possible by the learner’s perception and memory of regular sequential associations. We also find evidence of system-internal influence when we speculated that one form of *before* adverbial constructions led to regression in the accurate production of *without* constructions. These findings are not surprising given that complex systems are subject to influence from sources both internal and external to the system.

Although focused on the development of physical systems, Prigogine and Stengers (1984) suggest that “external fields… can be “perceived” by the system, creating the possibility of pattern selection” (p. 163). Clearly, the external “field” of the L2 ecology was perceived through continued exposure, resulting in competition between both contextually dominant and non-dominant forms. Unlike bifurcations in L1 development, however, the competition present between major form alternatives in the L2 is characterized not by acceptable, ecologically dominant forms (e.g., *before coming to the United States* vs. *before I came to the United States*), rather, by major form alternatives that represent both ecologically dominant as well as non-dominant forms, traditionally understood as errors. The subsequent pruning of certain forms from the linguistic repertoire is telling of the role of ecological pressure on L2 development. Whereas acceptable major form alternatives in English would presumably both continue to persist within the speaker’s repertoire, this is not the case in the L2 analysis presented above. The lack of availability of the divergent L2 forms in the usage patterns of the ambient language results in a precipitation of these forms out of the user’s language. Essentially, non-dominant forms are pruned from the L2 repertoire much in the same way that underdeveloped neuronal connections are pruned as the child develops cognitively (Webb et al., 2001).

### Competition, Pruning, and Form Alternatives in L2 Development

During periods of bifurcation characterized by heightened competition between major form alternatives, the language user is confronted with multiple equilibrium solutions, or attractor states, that govern meaningful production at any given time. Indeed, as MacWhinney (2015) argues, “individuals must continuously make choices between alternative ways of expressing intentions” (p. 10). This choice of expression is illustrated in the trajectories of Alceste’s development at the onset of bifurcation as bimodality in production was witnessed between both types of adverbial constructions. Discussion as to what motivates a language learner to recall one form and not another is speculative; however, it seems plausible that regression to earlier divergent forms, even when the learner has demonstrated more contextually convergent usage, may be due to the effect of hysteresis inherent to the system. In this way, changes in certain psychoemotional variables (e.g., anxiety, fatigue, distraction, and stress, etc.) may motivate regression to earlier states. Additionally, as linguistic resources are not homogeneous, the learner may agentively retain earlier contextually divergent forms to meet his needs for greater social proximity and conformity with or distance from his interlocutor at the time.

Though hysteresis spawned instances of bimodal regression in Alceste’s production over a period of several weeks, as the new attractors grew increasingly stable, the contextually divergent forms were eventually pruned from production in the data collected for this study. Not unlike the neuroanatomical changes that occur in late childhood and adolescence, characterized by the environmentally regulated elimination of “inappropriate synapses and their branches” (Webb et al., 2001, p. 157), the pressure from the L2 context mirrors a similar process of de-motivating the selection of divergent forms. Though these forms may resurface spontaneously in future language use due to both hysteresis and the heterogeneity of linguistic resources, the iterative reinforcement of convergent forms results in an increasing preference for their selection.

One interpretation of such pruning in L2 development rests on the assumption that the language user’s adaptation to the linguistic environment is a strong motivator of change within the system (Larsen-Freeman, 2006b). In this way, the contextually bound language use in which the L2 user engages promotes a process of adaptation that is not a strictly linear transition from contextually non-dominant to dominant forms. Much akin to the speciation and extinction of biological life forms, the cladistic branching of linguistic structures results in the exaptation of the L1 pattern (Gould and Vrba, 1982), the adaptation of those usage patterns readily perceptible in the environment, and the pruning, or selective suppression, of those which are not.

## Conclusion

In this article, data demonstrating bifurcations in the development of L2 syntax were analyzed from an untutored learner of English as a second language. This approach allowed for the qualitative features associated with the bifurcations to be scrutinized, thus detailing the emergence and restructuring of the attractor states governing the production of two syntactic constructions. The method of bifurcation analysis proved effective in uncovering the emergence of these forms, though the amount of data required to expose these patterns is indeed formidable. Further collaboration among CDST-oriented researchers on datasets with greater duration and density may add to our understanding of bifurcations and the significance that the patterns in the flux hold for L2 development.

If the bifurcation pattern of emergence holds true for other structures and contexts, it is supportive of a reconceptualization of the notion of error and dysfluency. Traditional models of proficiency presuppose gradual attenuation of these features as learners progress from one conceptual level of proficiency to the next. If the bifurcations spawned from the competition of syntactic forms are truly the “milestones” (Plaza-Pust, 2008) of language development, it is reasonable to assume that the heightened dysfluency and inaccuracy associated with these periods of instability are actually indicative of growth and not regression as intuition would suggest. This idea, of course, is highly amenable to our understanding of development from a complexity perspective.

The analysis of bifurcations presented here extends this understanding. Although overall growth in accuracy and fluency may be evident within a dataset, heightened variability associated with these constructs may be indicative of those moments in which linguistic knowledge passes through bifurcations and eventually converges on new orders of complexity. This notion clearly echoes Prigogine and Stengers (1984) “order through fluctuation” (p. 178). Attempting to view language development, particularly as it regards complex syntax, as periods of bifurcation is distinctly reminiscent of the way in which fluctuation, or oscillations within state space, ultimately leads to conceptually higher levels of order. Importantly, the significance of bifurcations in language development strongly reaffirms the position that not only should variation be acknowledged, but also that it is indispensable to development (e.g., Ellis, 1985; van Dijk et al., 2011). As Kelso (2018) put it, “variability is crucial for exploring the repertoire of states of a system and for taking the system into new territory” (n.p.). Clearly, studying such variation provides a critical window into the development of human behavior (Thelen and Smith, 1994; de Bot et al., 2007, de Bot, 2015; van Geert, 2008; Lowie, 2017).

In sum, the changing relationships between accuracy and fluency over time may indeed be explained endogenously by dynamic competition for attentional resources (Spoelman and Verspoor, 2010) and/or, exogenously, from the first order affordances (Larsen-Freeman, 2016) and constraints of the L2 ecology. Periods of greater competition between grammatical forms may be indicative of the restructuring of underlying concepts, or self-organization, and, as such, merit more detailed consideration of how these processes unfold over time. At the moment of bifurcation, i.e., the “edge of chaos” (Kauffman, 1995), the instability associated with the transition from one local attractor state to the next likely occasions certain regressions in performance in connected, more global, areas of competency—a consequence of the sensitive dependence on initial conditions, or the butterfly effect (Lorenz, 1963), that governs the development of complex systems. The final path that second language development appears to follow, it seems, is indelibly linked to system internal and system-environment interactions—a concept which has clearly resonated within discussions of language as a complex system (Larsen-Freeman and Cameron, 2008; de Bot, 2015; Lowie and Verspoor, 2015).

## Data Availability Statement

The raw data supporting the conclusions of this article will be made available by the authors, without undue reservation.

## Ethics Statement

The studies involving human participants were reviewed and approved by the University at Buffalo, Institutional Review Board. The patients/participants provided their written informed consent to participate in this study.

## Author Contributions

Both authors listed have made a substantial, direct and intellectual contribution to the work, and approved it for publication.

## Conflict of Interest

The authors declare that the research was conducted in the absence of any commercial or financial relationships that could be construed as a potential conflict of interest.

## Footnotes

1 By *learner’s system*, we refer to the current state of the learner’s linguistic repertoire.

2 We employ the term relexification as a deliberate attempt to move away from the notion of negative transfer which has come to be seen with increasing disfavor in the field of SLA. Relexification not only agentivizes the learner in the developmental process, but also destigmatizes “negative” transfer as something unfavorable or adverse when, in fact, this process may permit successful communication. Given its original distinction in Bickerton (1977) and Schumann (1981), relexification occurs not just with lexical items, but with syntactic constructions as well.

3 Moving away from problematic definitions of accuracy as the conformity to native-speaker norms (Larsen-Freeman and Evans, 2019), in this article, we understand (in-) accuracy as the divergence from/convergence toward a contextually dominant form as verified by linguistic corpora. As such, contextually dominant/non-dominant and contextually convergent/divergent are used interchangeably.
